# Sickling Cells, Cyclic Nucleotides, and Protein Kinases: The Pathophysiology of Urogenital Disorders in Sickle Cell Anemia

**DOI:** 10.1155/2012/723520

**Published:** 2012-06-13

**Authors:** Mário Angelo Claudino, Kleber Yotsumoto Fertrin

**Affiliations:** ^1^Laboratory of Multidisciplinary Research, São Francisco University (USF), 12916-900 Bragança Paulista, SP, Brazil; ^2^Hematology and Hemotherapy Center, University of Campinas (UNICAMP), 13083-970 Campinas, SP, Brazil

## Abstract

Sickle cell anemia is one of the best studied inherited diseases, and despite being caused by a single point mutation in the *HBB* gene, multiple pleiotropic effects of the abnormal hemoglobin S production range from vaso-occlusive crisis, stroke, and pulmonary hypertension to osteonecrosis and leg ulcers. Urogenital function is not spared, and although priapism is most frequently remembered, other related clinical manifestations have been described, such as nocturia, enuresis, increased frequence of lower urinary tract infections, urinary incontinence, hypogonadism, and testicular infarction. Studies on sickle cell vaso-occlusion and priapism using both *in vitro* and *in vivo* models have shed light on the pathogenesis of some of these events. The authors review what is known about the deleterious effects of sickling on the genitourinary tract and how the role of cyclic nucleotides signaling and protein kinases may help understand the pathophysiology underlying these manifestations and develop novel therapies in the setting of urogenital disorders in sickle cell disease.

## 1. Introduction

 Sickle cell anemia (SCA) has been first described over a century ago [1] and has become one of the best studied inherited human diseases. Despite being caused by a single point mutation in the *HBB *gene, multiple pleiotropic effects of the abnormal hemoglobin S production range from vaso-occlusive crisis, stroke, and pulmonary hypertension to osteonecrosis and leg ulcers [2–4].

Genitourinary tract function is also affected in SCA, and although priapism is most frequently remembered, other related clinical manifestations have been described, such as nocturia, enuresis, increased frequency of lower urinary tract infections, urinary incontinence, hypogonadism, and testicular infarction. Sickle hemoglobin S (HbS) polymerizes when deoxygenated, resulting in a series of cellular alterations in red cell morphology and function that shorten the red cell life span and lead to vascular occlusion. Sickle cell disease (SCD) vaso-occlusion constitutes a complex multifactorial process characterized by oxidative stress and recurrent ischemia-reperfusion injury in a vicious circle contributing to reduced blood flow and results, eventually, in complete obstruction of the microcirculation and organic dysfunction [3–6]. The exact pathogenetic mechanisms that tie genitourinary complications to the fundamental event of HbS polymerization and hemolytic anemia in SCA have just about started to be unraveled.

This paper focuses on how previous, sometimes poorly explained, clinical observations of urogenital disorders in patients with SCD relate to more recent discoveries on the role of cyclic nucleotides and protein kinases in the pathophysiology of sickle vaso-occlusion. 

## 2. Priapism

Priapism is defined as a prolonged and persistent penile erection, unassociated with sexual interest or stimulation, and is one of the complications associated with sickle cell anemia (SCA) since early in 1934 [7]. Priapism reaches a frequency of up to 45% in male patients with SCA, and the rate of resulting erectile dysfunction (ED) exceeds 30% [8–10]. Although this complication has been previously reviewed in depth in this journal [11], the main concepts behind its pathophysiology will be summarized here for better understanding of the mechanisms discussed throughout the paper, but readers are encouraged to read the previous review.

According to the American Urological Association Guidelines on the Management of Priapism, priapism can be subdivided into three categories: ischemic, stuttering, and nonischemic. Ischemic priapism (veno-occlusive, low flow) is a persistent erection marked by rigidity of the corpora cavernosa (CC) and little or no cavernous arterial inflow. In ischemic priapism, there are time-dependent changes in the corporal metabolic environment with progressive hypoxia, hypercarbia, and acidosis that typically generate penile pain. Penile sinusoids are regions prone to red blood cell sickling in SCD men because of blood stasis and slow flow rates, and ischemic priapism is thought to result from prolonged blockage of venous outflow by the vaso-occlusive process. Clinically, there is congestion and tenderness in the CC, sparing the glans and corpus spongiosum, usually with a prolonged course of over 3 hours, and frequently resulting in fibrosis and erectile dysfunction. Stuttering priapism (acute, intermittent, recurrent ischemic priapism) is characterized by a pattern of recurrence, but an increasing frequency or duration of stuttering episodes may herald a major ischemic priapism. Nonischemic priapism (arterial, high flow) is a persistent erection caused by unregulated cavernous arterial inflow. Typically, the corpora are tumescent but not rigid, the penis is not painful and is most frequently associated with trauma [12–16].

Conventional treatments are largely symptomatic, usually administered after the episode of priapism has already occurred, because the etiology and mechanisms involved in the development of priapism are poorly characterized [17, 18]. Preventive interventions have been proposed but, without a clear idea of the molecular mechanisms involved, they remain largely impractical to be applied in a regular basis in the clinic [17]. Due to the difficulty in exploring these mechanisms in patients, the use of animal models of priapism has become of utmost importance to decipher this devastating clinical challenge [19]. Animal models for priapism include dogs [20, 21], rabbits [22], rats [23–27], and mice [28–41].

Molecular biology and genetic engineering have been widely used in animal models to explore gene function in both human physiology and in the study of pathology of human priapism. Four major priapism animal models have been developed and have yielded greater knowledge on the intrinsic mechanisms underlying priapism: the intracorporal opiorphins gene transfer rat model [42–45], the endothelial nitric oxide synthase (eNOS) with or without neuronal NOS (nNOS) knock-out (eNOS^−/−^ ± nNOS^−/−^) mouse models [28, 29, 31–33], the adenosine deaminase knock-out (Ada^−/−^) mouse model [35, 36, 40, 41] and the transgenic sickle cell Berkeley mouse model [30, 33, 34, 37–39]. However, the Berkeley mouse is the only well-accepted animal model that displays clinical manifestations similar to those seen in humans with severe forms of SCD, including priapism [30, 34].

Priapism is essentially a derangement of normal erection. Penile erection is a hemodynamic event that is regulated by smooth muscle relaxation/contraction of corpora cavernosa and associated arterioles during sexual stimulation. The penile flaccidity (detumescence state) is mainly maintained by tonic release of norepinephrine through the sympathetic innervations of vascular and cavernosal smooth muscle cells [46]. During penile erection (tumescence state), vascular smooth muscle relaxation decreases vascular resistance, thereby increasing blood flow through cavernous and helicine arteries and filling sinusoids, which are expanded due to the relaxation of smooth muscle cells in the CC [47]. This physiological relaxation of penile smooth muscle is mainly, although not solely, mediated by the neurotransmitter nitric oxide (NO) that is produced by enzymes called NO synthases (NOS). NOSs are subdivided into three isoforms, endothelial NOS (eNOS or NOS3), neural NOS (nNOS or NOS1), and inducible NOS (iNOS or NOS2) [48, 49]. In the penile smooth muscle, NO is released from both nitrergic nerves and the sinusoidal endothelium [46, 50–52]. NO stimulates the soluble guanylyl cyclase (sGC) in the cavernosal smooth muscle, triggering increased synthesis of cyclic GMP (cGMP) that provides the main signal for smooth muscle relaxation [53]. cGMP levels in the CC are regulated by the rate of synthesis determined by sGC and the rate of cGMP hydrolysis mediated by phosphodiesterase type 5 (PDE5) [54, 55]. It has been reported that plasma hemoglobin released by intravascularly hemolysed sickle erythrocytes consumes NO, reducing its bioavailability in the erectile tissue, skewing the normal balance of smooth muscle tone towards vasoconstriction [17, 56, 57]. Champion and collaborators [33] showed that the penile smooth muscle of SCD transgenic mice presents with dysregulated PDE5A expression activity. Moreover, these mice had spontaneous priapism, amplified CC relaxation response mediated by the NO-cGMP signaling pathway, and increased intracavernosal pressure *in vivo *[37, 38].

Recent evidence has shown that another signaling pathway that may also contribute to the pathophysiology of priapism in SCD involves adenosine regulation. Similarly to NO, adenosine is a potent vasodilator produced by adenine nucleotide degradation. Adenosine is predominantly generated by adenosine monophosphate (AMP) dephosphorylation catalyzed by intracellular 5′-nucleotidase. Hydrolysis of s-adenosyl-homocysteine also contributes to intracellular adenosine formation [58, 59]. Extracellular adenosine may be generated by both adenine nucleotide degradation and dephosphorylation by ectonucleotidases [60]. Adenosine is then catabolized by two enzymes: adenosine kinase (ADK), which phosphorylates adenosine to AMP and is an important regulator of intracellular adenosine levels; and adenosine deaminase (ADA), which catalyzes the irreversible conversion of adenosine to inosine [58].

Several physiological processes may be affected by extracellular adenosine and this is mediated by four different receptors, referred to as A_1_, A_2A_, A_2B_, and A_3_. All four subtypes are members of the G protein-coupled receptor (GPCR) superfamily. The activation of the A_1_ and A_3_ adenosine receptors inhibits adenylyl cyclase activity and also results in increased activity of phospholipase C, while activation of the A_2A_ and A_2B_ subtypes increases adenylyl cyclase activity [58, 61]. Adenosine-induced vasodilation is mediated by increasing intracellular cyclic adenosine monophosphate (cAMP) levels in vascular smooth muscle cells via A_2_ receptor signaling [62, 63]. cAMP activates protein kinase A (PKA) resulting in decreased calcium-calmodulin-dependent MLC phosphorylation and enhanced smooth muscle relaxation [64]. Its role in penile erection has been investigated in studies showing that intracavernous injection of adenosine resulted in tumescence and penile erection [36, 61, 65]. In addition, adenosine induces NO synthesis in endothelial cells through A_2_ receptor signaling, and adenosine-mediated CC relaxation is partially dependent on endothelium-derived NO [36, 66–70].

A priapic phenotype in Ada^−/−^ mice was identified and led to further investigation of the impact of adenosine in the pathophysiology of priapism [59]. Previous reports showed that high levels of adenosine caused prolonged corporal smooth muscle relaxation *in vitro*. However, this effect was quickly corrected by intraperitoneal injection of a high dose of polyethylene glycol-ADA (PEG-ADA), which effectively reduces adenosine levels systemically [36, 71]. Moreover, adenosine induced significant increases in cavernosal cAMP levels via A_2B_ receptor activation. This demonstrated that A_2B_ receptor signaling is required for adenosine-mediated stimulation of cAMP production in CC smooth muscle cells [36, 71]. Mi and collaborators [36] have studied adenosine levels in the penis of sickle cell mice and have found a significant increase in adenosine levels, suggesting that overproduction of adenosine may contribute to priapic activity in SCD [71, 72]. Sickle cell mice submitted to PEG-ADA treatment suffered significant reduction of force and duration of relaxation when compared with untreated mice [71]. In addition, increased adenosine levels contributed to the development of penile fibrosis in Ada^−/−^ mice as well as in transgenic sickle cell mice [72]. These findings suggest a general contributory role of elevated adenosine in the pathophysiology of priapism associated with SCD. 

Although the penile vascular endothelium and smooth muscle cells are sources of vasodilation factors such as NO and adenosine, there are vasoconstriction pathways important to the penile hemodynamics, such as the Rho-kinase (ROCK) pathway. The RhoA/ROCK signal transduction pathway has been shown to influence erectile function *in vivo* through an array of mechanisms, including vasoconstriction of the penile vasculature via smooth muscle contraction and regulation of eNOS [73–76]. This pathway is involved in the regulation of smooth muscle tone by modulating the sensitivity of contractile proteins to Ca^2+^ [77]. RhoA regulates smooth muscle contraction by cycling between a GDP-bound inactive form (coupled to a guanine dissociation inhibitor, RhoGDI) and a GTP-bound active form [78–80]. Upstream activation of heterotrimeric G proteins leads to the exchange of GDP for GTP, an event carried out by the guanine exchange factors (GEFs) p115RhoGEF [81], PDZ-RhoGEF [82], and LARG (Leukemia-associated RhoGEF) [83], which are able to transduce signals from G protein-coupled receptors to RhoA [84–86]. ROCK is activated by RhoA and inhibits myosin phosphatase through the phosphorylation of its myosin-binding subunit, leading to an increase in Ca^2+^ sensitivity. The RhoA/ROCK Ca^2+^ sensitization pathway has been implicated in the regulation of penile smooth muscle contraction and tone both in humans and animals [77, 87]. ROCK exerts contractile effects in the penis by Ca^2+^-independent promotion of myosin light chain (MLC) kinase or the attenuation of MLC phosphatase activity and reduction in endothelial-derived NO production [88]. RhoA activation, ROCK2 protein expression, as well as total ROCK activity decline in penile of SCD transgenic mice, highlighting that the molecular mechanism of priapism in SCD is associated with decreased vasoconstrictor activity in the penis [39]. Therefore, should impaired RhoA/ROCK-mediated vasoconstriction contribute to SCD-associated priapism, this pathway may become a novel therapeutic target in the management of this complication.

There has been no definite advance in the management of sickle cell-associated acute, severe priapism. Penile aspiration with or without saline intracavernosal injection and eventually performing surgical shunts remains mainstays of care, with no evident benefit of more common approaches, such as intravenous hydration, blood transfusions, and urinary alkalinization [89, 90]. Pharmacological interventions in such cases have been limited to intracavernosal use of sympathomimetic drugs, such as epinephrine, norepinephrine, and etilefrine, but there are anecdotal reports of acute use of PDE5 inhibitor sildenafil [91].

Nonetheless, most attempts to control SCD priapism have focused on its recurrent, stuttering form. Small case series of hormonal manipulation with diethylstilbestrol [92], gonadotropin-releasing hormone (GnRH) analogues [93], and finasteride [94] have been reported to successfully manage recurrent priapism. Increasing smooth muscle tone with oral *α*-agonist etilefrine has also yielded only anecdotal evidence of benefit [95]. Unfortunately, a prospective study comparing etilefrine and ephedrine failed to demonstrate superiority or equivalence of both drugs in preventing recurrent priapism due to poor compliance and low recruitment reducing statistical power, but some evidence was obtained reassuring safety of the use of such strategies, and possibly indicating a lower severity of priapism attacks among compliant patients [96]. This favors off-label use of pseudoephedrine at bedtime advocated by some experts [57, 90]. Hydroxyurea has also been effective in preventing priapism recurrence in SCD in a small number of cases [97, 98]. Based on current knowledge of NO-dependent pathways, the use of PDE5 inhibitors has been studied. One clinical trial testing tadalafil in SCD patients has been terminated, but no outcome data have yet been published (ClinicalTrials.gov NCT00538564), and one ongoing trial aims at the effect of sildenafil in the same setting (ClinicalTrials.gov NCT00940901). Despite these efforts, scientists have become less optimistic concerning the tolerability of this approach, ever since the premature termination of the sildenafil trial for pulmonary hypertension in SCD patients, in which subjects on PDE5 inhibitor were more likely to have severe pain crises requiring hospitalization [99]. Therefore, novel therapies for preventing and treating priapism in SCD are still warranted if the incidence of impotence among these patients is expected to be reduced in the long term.

## 3. Infertility

Progress in the therapy of SCD, particularly the use of hydroxyurea, has considerably improved the prognosis of patients with SCD [100, 101], with their mean life expectancy reaching much over 40 years [102–104], rendering infertility an important issue. Nevertheless, long before hydroxyurea became a standard of care in SCD, seminal fluid parameters of SCD males had been reported to fall within the subfertile range due to decreased sperm concentration, total count, motility, and altered morphology [105–107], and a more recent study reported over 90% of patients had at least one abnormal sperm parameter [108].

Hydroxyurea (HU) has been reported to impair spermatogenesis, causing testicular atrophy, reversible decrease in sperm count, as well as abnormal sperm morphology and motility [108–114], and its current or previous use should be among the first probable causes to be considered in SCD patients complaining of infertility. Moreover, sperm abnormalities prior to HU have been attributed to variable effects of hypogonadism induced by SCD itself, and lack of appropriate testosterone production seems to be exacerbated by HU use in a mouse SCD model [115].

Considering that male fertility does not rely solely on the quality of the seminal fluid, other causes that may also render male patients with SCD prone to suffer from infertility include sexual problems, such as loss of libido, premature ejaculation, frequent priapism, and priapism-related impotence [105–107, 116–121].

Finding a single main cause for male infertility in a particular SCD patient is highly unlikely and probably will involve some degree of endocrinological impairment. A broader understanding of how hypogonadism takes place in SCD is necessary to explain fertility problems and requires knowledge of the complexity of sex hormone production regulation.

## 4. Hypogonadism

The etiology of hypogonadism in SCD patients is multifactorial, as several mechanisms have been suggested to contribute to its occurrence, such as primary gonadal failure [117, 122, 123], associated with or caused by repeated testicular infarction [124], zinc deficiency [125, 126], and partial hypothalamic hypogonadism [127].

Physical and sexual development are affected in both male and female SCD patients, with onset of puberty (menarche) and appearance of secondary sexual characteristics (pubic and axillary hair and beard) being usually delayed. The delay is greater in homozygous SCA and S-*β* ^0^-thalassemia than in SC disease and S-*β* ^+^-thalassemia [128–130]. Moreover, studies in male patients with SCD reported reduction of ejaculate volume, spermatozoa count, motility, and abnormal sperm morphology [106, 116].

Biochemical analyses have demonstrated low levels of testosterone and dihydrotestosterone and variable levels of follicle-stimulating hormone (FSH) and luteinizing hormone (LH) in patients with SCD [105–107, 118, 119, 121, 131]. The comparison between patients and controls matched according to stage of development of secondary sexual characteristics showed higher levels of LH in sickle cell disease, favoring some role for hypergonadotropic hypogonadism.

Leydig cells of the testes and other steroidogenic tissues produce hormones by a multienzymatic process, in which free cholesterol from intracellular stores is transferred to the outer and then to the inner mitochondrial membrane. Leydig cells produce androgens under the control of LH or its placental counterpart human chorionic gonadotropin (hCG), as well as in response to numerous intratesticular factors [114, 132]. LH/hCG receptors belong to the sGC-coupled seven-transmembrane-domain receptor family, whose activation leads to stimulation of adenylyl cyclase [133]. The resulting accumulation of intracellular cyclic adenosine monophosphate (cAMP) levels and the concomitant activation of the cAMP-dependent protein kinase (PKA) lead to the phosphorylation of numerous proteins, including the steroidogenic acute regulatory (StAR) protein [134, 135]. StAR localizes predominantly to steroid hormone-producing tissues and consists of a 37 kDa precursor containing an NH2-terminal mitochondrial targeting sequence and several isoelectric 30 kDa mature protein forms [136–138]. Steroid production in gonadal and adrenal cells requires both *de novo* synthesis and PKA-dependent phosphorylation of StAR-37 protein [139]. The newly synthesized StAR is functional and plays a critical role in the transfer of cholesterol from the outer to the inner mitochondrial membrane, whereas mitochondrial import and processing to 30 kDa StAR protein terminate this action [140–142].

HbS polymerization is mediated by upstream activation of adenosine receptor A_2B_R by hypoxia, and hemolysis of irreversibly sickled red blood cells increases adenosine bioavailability through conversion of ATP by ectonucleotidases CD39 and CD73, thus predisposing patients with SCD to sustained high levels of cAMP [143, 144]. From this point of view, steroidogenesis could be expected to be increased in these patients.

Although Leydig cell steroidogenesis is predominantly regulated by cAMP/PKA, other pathways also influence this process [145], including the NO-cGMP signaling pathway [146]. NO promotes a biphasic modulation in the androgen production, stimulatory at low concentrations, and inhibitory at high concentrations [49, 147, 148]. SCA causes NO depletion, and in low levels, NO stimulates Leydig cell steroidogenesis by activating sGC [48, 49, 149] and promotes the formation of low levels of cGMP, albeit enough to activate the cGMP-dependent protein kinase (PKG) and phosphorylate StAR [49, 150]. This signaling is controlled by phosphodiesterases (PDEs) [151] and active transport systems that export cyclic nucleotides (multidrug-resistance proteins) from the cell [152]. In zona glomerulosa cells, activation of PKG II by cGMP regulates basal levels of aldosterone production and phosphorylation of StAR protein [150], but whether there is a role for cGMP in the zona reticularis, where adrenal androgenesis takes place, is unknown.

**Figure 1:**
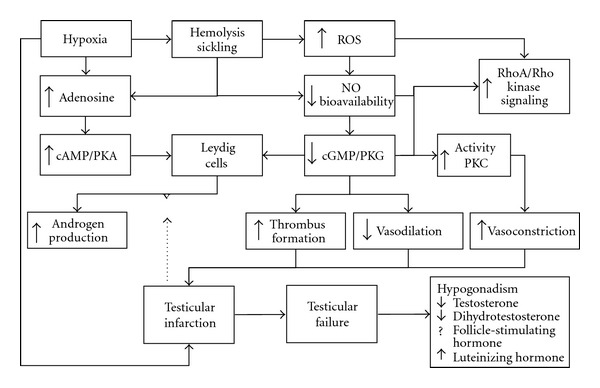
Schematic pathophysiology of hypogonadism and testicular infarction in sickle cell disease. The dashed arrow represents the blocking effect of gonadal failure over cyclic nucleotide-stimulated androgen production.

Hypogonadism observed in patients with SCD with lower circulating testosterone and higher LH levels suggests that, at least in this setting, despite the reduced cGMP- and elevated cAMP-mediated stimuli on androgen production, gonadal failure with Leydig cell impairment predominates in sex hormone production dysfunction (Figure 1). This further highlights that primary hypogonadism is possibly largely underdiagnosed and elicits more studies on the pathogenesis of testicular infarction.

## 5. Testicular Infarction

Segmental testicular infarction is an infrequent cause of acute scrotum and is rarely reported, with fewer than 40 cases published at the time of this paper. Its etiology is not always well defined, and it may be, at first, clinically mistaken for a testicular tumour [153, 154]. Common causes for testicular infarction are torsion of the spermatic cord, incarcerated hernia, infection, trauma, and vasculitis [131]. The usual presentation is a painful testicular mass unresponsive to antibiotics [155]. This testicular disorder has been associated with epididymitis, hypersensitivity angiitis, intimal fibroplasia of the spermatic cord arteries, polycythemia, anticoagulant use, benign testicular tumors and, in the interest of this review, sickle cell trait and sickle cell disease [124, 131, 155–158].

Testicular infarction related to sickling has been very rarely reported with only five individual cases found retrospectively, three associated with sickle cell disease and two with sickle cell trait [124, 155–157, 159]. Holmes and Kane reported the first testicular infarction in a patient with SCD who presented with testicular swelling unresponsive to antibiotics. Physical examination revealed that a lesion suspicious for malignancy and ultrasonography demonstrated a hyperechoic mass with an anechoid rim and normal blood flow in the surrounding parenchyma. Radical orchiectomy revealed hemorrhagic infarction with sickle blood red cells. In another case report, SCA patient presented with acute scrotum and history of acute chest syndrome, splenic infarction, osteomyelitis, and hemolysis. Physical examination demonstrated an erythematous, tender, swollen testicle and ultrasound once again revealed normal echotexture and blood flow. Surgical exploration and pathological examination diagnosed segmental testicular infarction with vascular congestion and sickled red blood cells [124]. In the last testicular infarction case report in a patient with SCD presented with increased testicular volume, scrotal ultrasonography showed both echogenic and hypoechogenic regions and Doppler ultrasonography revealed vascular changes compatible with testicular infarction. Radical orchiectomy was performed 10 days after the initial presentation and microscopic evaluation showed necrotic seminiferous tubules devoid of nuclear debris, congestion, or acute inflammatory infiltrate, consistent with coagulative necrosis of ischemic origin [131].

Testicular blood flow is dependent on the internal spermatic, cremasteric, and deferential arteries. Obstruction of venous outflow may create venous thrombosis, testicular engorgement, and subsequent hemorrhagic infarction. In SCD, low oxygen tensions in erythrocytes lead to sickling cells that lose pliability in the microcirculation. Consequently, capillary flow becomes obstructed, worsening local tissue hypoxia, perpetuating the cycle of sickling, and promoting testicular infarction [124, 131, 157].

The cyclic nucleotides and protein kinases may play an important role in the pathophysiology of testicular infarction in SCD. Enhanced hemolysis and oxidative stress contribute to a reduction in nitric oxide (NO) bioavailability due to NO scavenging by free hemoglobin and reactive oxygen species (ROS) generation [160, 161]. As mentioned before, testicular NO signaling pathway is involved in the regulation of Leydig cell steroidogenesis [48, 49, 147–149, 162–164] but may also influence testicular circulation. We suggest that the reduction of NO bioavailability and consequent reduction of GMPc levels and of activity of PKG may decrease the vasodilation process in the testes. Moreover, reduced NO levels in patients with sickle cell disease contribute to the development of thrombus formation in the vascular system and could further enhance local ischemia [165, 166]. Furthermore, the cGMP-dependent protein kinase signaling pathway would normally inhibit RhoA-induced Ca^2+^ sensitization, RhoA/ROCK signaling, and protein kinase C (PKC) activity that mediate contraction in vascular smooth muscle [167–171]. Thus, reduced NO levels may decrease cGMP-dependent protein kinase activity and promote increasing RhoA-induced Ca^2+^ sensitization and PKC activity, favoring vasoconstriction in the testes. Therefore, tissue hypoxia, sickling of red blood cells, reduced levels of NO, possible thrombus formation, increased RhoA-induced Ca^2+^ sensitization, and PKC activity may all lead to capillary and venous flow obstruction promoting testicular infarction (Figure 1).

Although testicular infarction in SCD has been very rarely reported, it has been speculated that silent testicular infarctions are much more common but generally overlooked clinically. Testicular biopsy in patients is rarely performed and additional studies are necessary to establish the true incidence of testicular infarction in patients with SCD or even sickle cell trait.

## 6. Urinary Bladder Dysfunction

The urinary bladder has two important functions: urine storage and emptying. Urine storage occurs at low pressure, implying that the bladder relaxes during the filling phase. Disturbances of the storage function may result in lower urinary tract symptoms (LUTSs), such as urgency, increased frequency, and urge incontinence, the components of the hypoactive or overactive bladder syndromes [172, 173]. The passive phase of bladder filling allows an increase in volume at a low intravesical pressure. The bladder neck and urethra remain in a tonic state to prevent leakage, thus maintaining urinary continence. Bladder emptying is accompanied by a reversal of function in which detrusor smooth muscle (DSM) contraction predominates in the bladder body that is accompanied by a concomitant reduction in outlet resistance of the bladder neck and urethra [174–176]. The bladder filling and emptying are regulated by interactions of norepinephrine (sympathetic component released by hypogastric nerve stimulation), acetylcholine and ATP (parasympathetic components released by pelvic nerve stimulation) with activation of adrenergic, muscarinic, and purinergic receptors, respectively [175].

Urinary bladder dysfunction is rarely spontaneously reported by SCD patients to their caregivers. With increasing survival of these patients, physicians may expect that urinary complaints increase in association with classical urological disorders associated with advanced age, such as urinary stress incontinence in multiparous women and benign prostatic hyperplasia in men. Nonetheless, clinical observations of medical complaints involving the urinary bladder start as early as childhood, with enuresis, and continue onto adulthood with nocturia and urinary tract infections, to name a few, although frequently neglected.

Nocturia has long been attributed to constant increased urinary volumes in SCD. As part of the renal complications of sickling, renal medullary infarcts lead to decreased ability to concentrate urine, yielding higher daily urinary volumes [177], compensatory polydipsia, and eventually, the need for nocturnal bladder voiding.

For comparison, the effects of polyuria on bladder function have been better characterized in diabetic bladder dysfunction (DBD). Both SCD and diabetes mellitus cause increased urinary volume and, to some extent, the two diseases involve cellular damage by oxidative stress mediators; so data from previous studies on DBD may help shed some light on preliminary data on bladder function in SCD animal models by understanding a known model of bladder dysfunction.

It has been suggested that DBD comprehends so-called early and late phases of the disease, owing to cumulative effects of initial polyuria secondary to hyperglycemia, complicated by oxidative stress influence on the urothelium and nervous damage in the long term of the natural history of diabetes mellitus. In the early phase of DBD, the bladder is hyperactive, leading to LUTS comprised mainly by nocturia and urge incontinence. Later in the course of the disease, the detrusor smooth muscle becomes atonic, abnormally distended, and incontinence is mainly by overflow associated with a poor control of urethral sphincters, and voiding problems take over [178].

DSM physiology also involves cyclic nucleotides and activation of protein kinases. DSM contractions are a consequence of cholinergic-mediated contractions and decreased *β*-adrenoceptor-mediated relaxations [179]. DSM contains a heterogeneous population of muscarinic receptor subtypes [180, 181], with a predominance of the M2 subtype and a smaller population of M3 receptors. However, functional studies showed that M3 receptors are responsible for promotion of contraction in the DSM of several animal models [182–185] and in humans [186, 187]. Activation of M3 muscarinic receptors in the DSM promotes stimulation of phospholipase C, activates PKC, and increases formation of inositol trisphosphate (IP_3_) and diacylglycerol (DAG) to release calcium from intracellular stores, leading to DSM contraction [87]. Moreover, activation of M2 receptors also induces a DSM contraction indirectly by inhibiting the production of cAMP, reducing PKA activity, and reversing the relaxation induced by *β*-adrenoceptors [179]. Hence, both mechanisms promote urinary bladder emptying.

There is evidence that the Ca^2+^-independent RhoA/ROCK pathway is involved in the regulation of smooth muscle tone by altering the sensitivity of contractile proteins to Ca^2+^ [77]. This pathway has been shown to influence erectile function *in vivo* through an array of mechanisms, including phosphorylation of the myosin-binding subunit of MLC phosphatase, resulting in increased myosin phosphorylation. RhoA, a member of the Ras (Rat Sarcoma) low molecular weight of GTP-binding proteins, mediates agonist-induced activation of ROCK. The exchange of GDP for GTP on RhoA and translocation of RhoA from the cytosol to the membrane are markers of its activation and enable the downstream stimulation of various effectors such as ROCK, protein kinase N, phosphatidylinositol 3-kinase, and tyrosine phosphorylation [77]. The RhoA/ROCK Ca^2+^ sensitization pathway has been implicated in the regulation of bladder smooth muscle contraction and tone in humans and animals [77, 188–191]. Thus, alterations in the contraction or relaxation mechanisms of DSM during the filling and emptying phases may contribute to urinary bladder dysfunction. Patients with SCD have not been evaluated for bladder dysfunction in a systematic manner, but preliminary data have shown that Berkeley mice (homozygous SS) exhibit hypocontractile DSM *ex vivo*, due to a significant decrease of contractile responses to muscarinic agonist carbachol and electrical field stimulation [192]. This bladder dysfunction may contribute to the increased risk of urinary tract infections observed in SCD patients.

In an epidemiological study of 321 children with SCD, 7% had a documented urinary tract infection (UTI), one-third had recurrent infections, and two-thirds had had a febrile UTI [193]. As in normal children, there was a strong predominance of females, and gram-negative organisms, particularly *Escherichia coli*, were usually cultured. Most episodes of gram-negative septicemia in SCD are secondary to UTI [194]. Moreover, UTIs are more frequent during pregnancy in women with SCA or sickle cell trait [195–197]. The prevalence of UTI in women with SCA is nearly twofold that of unaffected black American women. This association appears to be directly related to HbS levels, since patients with sickle trait have an increased prevalence of bacteriuria, but to a lesser degree than those with SCA. More recently, a study detected that a group of SCD children and adolescents had more symptoms of overactive bladder than a control group [198]. This could be a first documentation of a clinically evident of an early phase of sickle cell bladder dysfunction, but whether there is a late, hypotonic bladder phase in older sickle cell adults remains to be demonstrated.

The presence of increased intracavernosal pressure associated with the amplified corpus cavernosum relaxation response (priapism) mediated by NO-cGMP signaling pathway, the lack of RhoA/ROCK-mediated vasoconstriction in sickle cell transgenic Berkeley mice, and the association of priapism with genitourinary infections and urinary retention further suggest the possibility that changes in the DSM reactivity may contribute to urogenital complications in SCD [36, 38–40, 192]. Despite advances in the understanding of urogenital disorders in the SCD, further studies should clarify the pathophysiological mechanisms that underlie genitourinary manifestations of SCD.

## 7. Conclusions

Urogenital disorders in SCD are the result of pleotropic effects of the production of the abnormal sickling hemoglobin S. While priapism still stands out as the most frequently encountered, current knowledge of the effects of cyclic nucleotide production and activation of protein kinases allows to suspect underdiagnosis of bladder dysfunction and hypogonadism secondary to testicular failure. Moreover, despite our growing understanding of these complications, adequate, efficacious, and well-tolerated treatments are still unavailable, and male patients continue to suffer from infertility and erectile dysfunction. Further work in, both clinical assessments and experimental studies in this field are promising and should help increase physicians' awareness of the importance of more accurate diagnoses, design improved therapeutic strategies, and eventually, achieve better quality of life for SCD patients.

## Abbreviations

ROS: Reactive oxygen species
NO: Nitric oxide
cAMP: Cyclic adenosine monophosphate
PKA: Cyclic adenosine monophosphate-dependent protein kinase
cGMP: Cyclic Guanosine monophosphate;
PKG: Cyclic Guanosine monophosphate protein kinase;
PKC: Protein kinase C.
